# A Synchronous Multi-Body Sensor Platform in a Wireless Body Sensor Network: Design and Implementation

**DOI:** 10.3390/s120810381

**Published:** 2012-07-31

**Authors:** Yeongjoon Gil, Wanqing Wu, Jungtae Lee

**Affiliations:** Graduate School of Computer Science and Engineering, Pusan National University, Pusan 609-735, Korea; E-Mails: kyzoon@pusan.ac.kr (Y.G.); wqwu@pusan.ac.kr (W.W.)

**Keywords:** EEG, ECG, PPG, respiration, multi-body sensor platform, synchronous body sensor

## Abstract

**Background:**

Human life can be further improved if diseases and disorders can be predicted before they become dangerous, by correctly recognizing signals from the human body, so in order to make disease detection more precise, various body-signals need to be measured simultaneously in a synchronized manner.

**Object:**

This research aims at developing an integrated system for measuring four signals (EEG, ECG, respiration, and PPG) and simultaneously producing synchronous signals on a Wireless Body Sensor Network.

**Design:**

We designed and implemented a platform for multiple bio-signals using Bluetooth communication.

**Results:**

First, we developed a prototype board and verified the signals from the sensor platform using frequency responses and quantities. Next, we designed and implemented a lightweight, ultra-compact, low cost, low power-consumption Printed Circuit Board.

**Conclusion:**

A synchronous multi-body sensor platform is expected to be very useful in telemedicine and emergency rescue scenarios. Furthermore, this system is expected to be able to analyze the mutual effects among body signals.

## 1. Introduction

In the United States, about 460,000 people die as a result of fatal heart attacks every year. Approximately half of these patients die within one hour of the start of symptoms, and before they can arrive at a hospital. To rectify this situation, many researchers are attempting to build a health care system that is faster and more accurate. In line with this trend, wireless body sensor network (WBSN) technologies have been developed, which are helping to improve the quality of human life [1]. In general, WBSNs contain multiple sensors for the measurement of various bio-signals on the body. These sensors enable abnormal signals to be detected via wireless communications, and thus emergency treatment can be applied more quickly [2].

The better the quality of life becomes, the more people become interested in their health. According to UK public spending data, the UK Gross Domestic Product (GDP) in 1985 was GBP 361.758 billion and the British spent GBP 19.4 billion on healthcare. In 2010, the UK GDP was GBP 1,453.62 billion, a 4.01-fold increase since 1985, but healthcare expenditure increased 6.09-fold to GBP 118.31 billion. Statistics such as these show that as the quality of life increases, more attention is paid to health, and more is spent on healthcare.

Even greater improvements to human life can be achieved if diseases and disorders can be predicted and treated before they become serious; this involves correctly reading signals from the human body. In accordance with this need, various colleges and research centers are actively involved in real-time healthcare research [2–5].

Research on the use of electroencephalography (EEG) to record brain activity, electrocardiography (ECG) to record heart activity, and photoplethysmography (PPG) to record the pulse has progressed, and many convenient instruments have been developed, even for use by non-medical staff [2–6].

However, a single body-signal measured in a non-invasive way indicates only a single aspect of a person's health. A human body has very intricate connecting systems, and so it is not appropriate to draw any specific conclusions from such data. Therefore, in order to draw more precise conclusions, various body signals need to be measured simultaneously.

Respiration has a direct influence on EEG, ECG, and PPG signals [7]. There is also a fairly high correlation between ECG and PPG. EEG is the most appropriate body-signal for gaining insights into various states of the human body such as emotions, tension, concentration, and relaxation. It is therefore expected that simultaneous recording of EEG variations and those of other body-signals would be very useful.

To this end, in this paper, we propose a synchronous multi-body sensor platform on a WBSN. Of necessity, the apparatus has low power-consumption, is low cost, has an ultra-compact design, and is lightweight because it will use the properties of each signal and measure the signals using one shared analog circuit. Above all, we designed the platform to be very convenient for use with a smartphone or a home or office PC. In the next section, we outline related works on WBSN. In Section 3, the design and architecture of each system are described, while the experimental setup and results for body signals obtained using this new apparatus are presented in Sections 4 and 5, respectively. The expected effects on and contributions to healthcare are described in Section 6.

## 2. Related Works on Wireless Body Sensor Network

The typical, requirements for WBSNs are as follows [1,8]:

- Reliability
- Low-power consumption
- Real-time processing
- Mobility
- Easy access and Comfort
- Small size and low-cost

Synchronized bio-signals are very useful in the analysis of some symptoms. However, even though efforts are made to measure them simultaneously with different apparatus, the signal measurements eventually become asynchronous. It is a complicated task to precisely analyze and draw conclusions from body-signals, and to establish correlations between the responses. Much research is therefore being conducted to develop a unitary apparatus that can both measure various kinds of signals simultaneously and yield synchronized signals. In 2010, Seoul National University of Technology (SNUT) in Korea developed and introduced equipment measuring ECG and PPG signals synchronously for more accurate non-invasive examination of blood-flow conditions in veins [3].

Our laboratory developed a portable eight channel EEG sensor platform based on IEEE 802.15.4 in 2009 [6]. This platform was designed with the sensor nodes and a Zigbee gateway (ZiGw) that consists of dual protocol stacks. The Zigbee protocol stack first receives the bio-signal data from the sensor nodes, after which it is transmitted to the Internet via the second (TCP/IP) protocol stack [6]. Thus, the ZiGw receives and relays the EEG data acquired from the sensor nodes. This arrangement is necessary for telemedicine and emergency medical care. Considering only a sensor node, the Zigbee communication is most advantageous in terms of power consumption. However, if no gateway such as ZiGw were available, then the sensor node would not work properly. These restrictions show that the mobility of Zigbee communication is very limited.

In a WBSN system, continuous data transmission must be guaranteed. An accurate analysis of bio-signals with errors is impossible. In addition, if bio-signals are compressed or aggregated, then real-time feedback is very difficult to implement. It should be noted that, the data loss rate of the Zigbee communication was higher in actual experiments. Therefore, we had to implement an interpolation algorithm to compensate for the data error/loss in the ZiGw. When the EEG recovered from this algorithm in the ZiGw and that from the original EEG source in the sensor node were compared, we found that the data were not identical.

Therefore, as the next best thing, we chose Bluetooth for wireless communication. Bluetooth is a wireless communication method that is built into most smartphones. Thus, if a smartphone is used with the system, the system designer does not need to develop additional relay equipment, such as ZiGw. In terms of cost, there is no need for ZiGw included dual protocol stack, therewith it takes a much cheaper cost. In addition, users could be easily connected to smartphones without a complicated set-up process.

Last year, we developed an ECG platform based on ATmega128L (Atmel Corporation., San Jose, CA, USA) using Bluetooth communication in our laboratory [9]. We then modified this platform in order to measure multi-bio signals. However, in order to use this platform to measure the four vital signs, too many op-amps and passive elements were needed. As a result, this platform consumed a lot of power in actual experiments. In this paper, we propose a more efficient sensor platform for the measurement of multi-bio signals in WBSN.

## 3. Design and Implementation

In this research, our goal is to obtain four kinds of body-signals (EEG, ECG, respiration, and PPG) using one integrated system. However, the measurement of each signal depends on different modalities especially in terms of low pass filter (LPF) and total gain as shown in Table 1, requiring the implementation of frequency range and gain [5,6,9].

As seen in Table 1, to avoid 60-Hz power-line noise in EEG and ECG measurements, a relatively high common mode rejection ratio (CMRR) is necessary, to reject common mode signals [6]. Although the high-pass-filter (HPF) bandwidths of all the body-signals are identical, the low-pass-filter (LPF) bandwidths vary, as do the benefits of each signal.

### 3.1. Common-Mode Rejection Ratio (CMRR)

**Figure 1.**
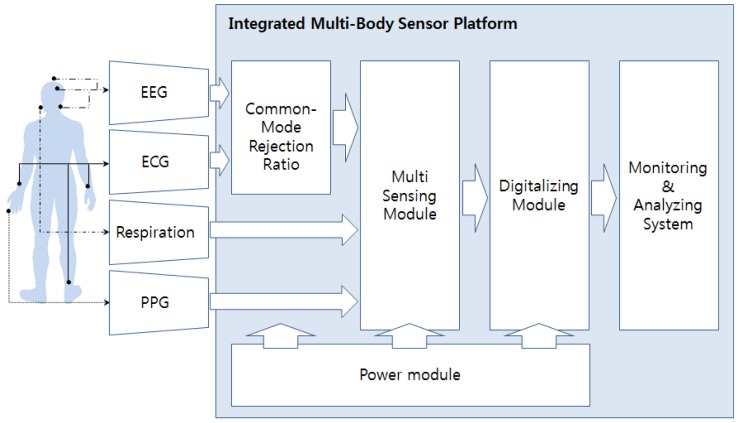
Structure of the integrated multi-body sensor platform.

**Figure 2.**
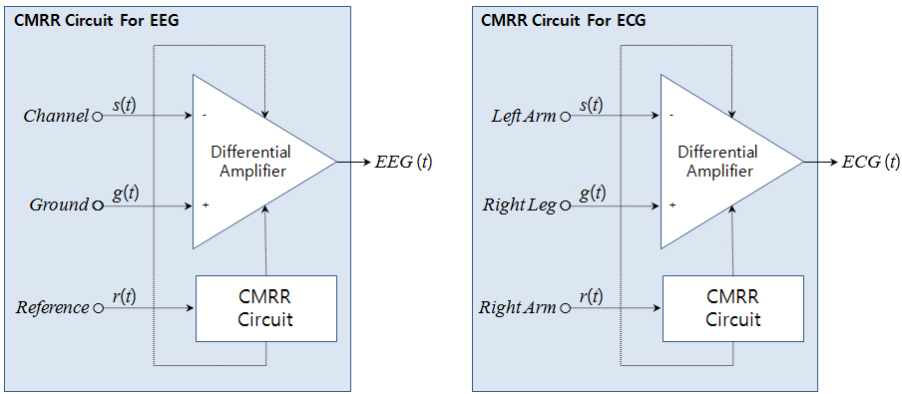
Structure of Common-mode rejection ratio for EEG and ECG.

As seen in Figure 1, in order to avoid 60-Hz power-line noise in measuring EEGs and ECGs, high CMRR is needed. According to Figure 2, in the case of EEG, the channel signal is *s(t)* and the ground signal is *g(t)*. Hence, the differential amplifier operates on the basis of the voltage difference of the two signals. At this point, *EEG(t)* uses a CMRR circuit with a reference signal (*r(t)*) to reduce noise. Therefore, three electrodes are required to measure EEG signals. In the case of ECG, the channel signal is *s(t)* from the left arm, the ground signal is *g(t)* from the right leg, and the reference signal is *r(t)* from the right arm.

A high input impedance, high CMRR, and moderately high gain instrumentation amplifier is a good choice as the differential amplifier for the EEG conditioning circuit [6]. For our work, we selected an AD620 amplifier chip (Analog Devices, Inc., Norwood, MA, USA); this chip has a variety of advantages such as low cost, low power-consumption, and low input bias current [10].

It also features a high CMRR of 120 dB and a differential input impedance of 10 GΩ‖2pF, which satisfies the required conditions well [5,6]. As seen in Figure 2, a CMRR circuit requires three-port inputs. EEG signals typically consist of one channel signal, one reference and one ground signal. However, the PPG and respiration signal does not need the CMRR circuit. It needs only a one-port direct input. The distinguished signals are first amplified using a differential amplifier [5,6,9]. The gain of this amplifier is 20 dB.

### 3.2. Design of Multi Sensing Module

From a general point of view, the *EEG (t) and ECG (t)* are first amplified by the CMRR module, undergo filtering in the multi-sensing module, and then go through a second amplifier. As shown in Table 1, the LPFs vary in accordance with the properties of each signal.

**Figure 3.**
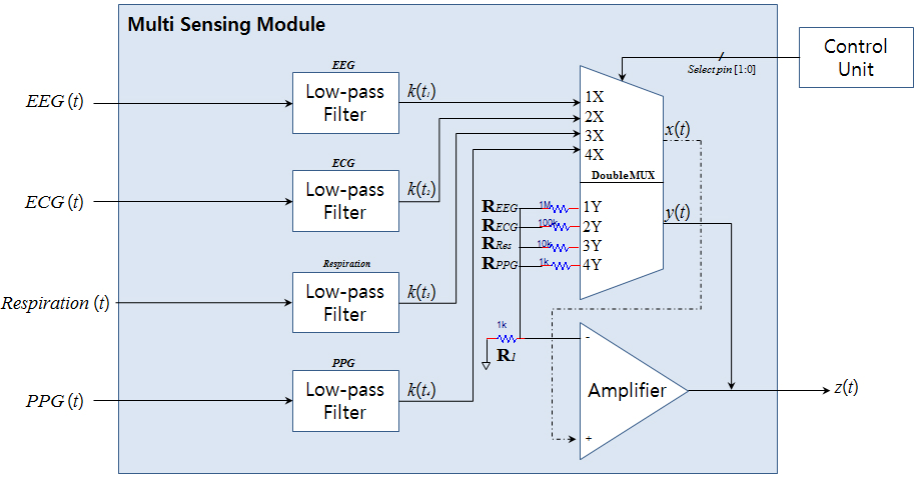
Structure of multi sensing module.

EEG needs a LPF of 50 Hz and a total gain of 80 dB. Because the *EEG (t)* signal has already undergone an amplification of 20 dB via the differential amplifier in CMRR, the amplifier for the multi-sensing module only requires an amplification of 60 dB. Thus, in Figure 3, R*1* is 1 kΩ and R*eeg* is 1 MΩ. In this module, the frequency of the LPF and the amplifications of the other signals (ECG, respiration, and PPG) are also designed according to the ratios and system specifications listed in Table 1.

After throughout the LPF, *k(t)* are determined by the figure gained through the Double 4 to 1 Multiplexer SN74HC4852N (Texas Instruments, USA) [11]. At this stage, *z(t)*, the final output, is the output signal in accordance with the amplification factor *y(t)*, which is determined by the *x(t)* signal. Also, the 2-bit select-pin [1:0] signal, which is generated for choosing signals, is synchronized with the control unit from MCU, which operates at the multi-sensing interface module and is transferred.

For example, when the MUX operates on the 1X and 1Y inputs, the EEG signal is amplified by an amplification of 60 dB, and when it operate on the 2X and 2Y inputs, the ECG signal is amplified by an amplification of 40 dB. MUX is used in order to reduce both the number of op-amps and the power consumed. These effects decrease overall cost and extend battery life.

### 3.3. Design of Digitalizing Module

The main processor used for digitalizing is an ATmega128L (Atmel Corporation, San Jose, CA, USA). This microcontroller has a 128-KByte in-system programmable flash, a 4-KByte EEPROM for information storage, and a 4-KByte Internal SRAM [12]. It also includes a 10-bit analog-to-digital converter (ADC).

The multiplexer chip operating in the previous stages causes minute delays in signal outputs, according to its properties. These delays are nullified by an ADC, which modulates the sampling rate in the digitalizing system.

**Figure 4.**
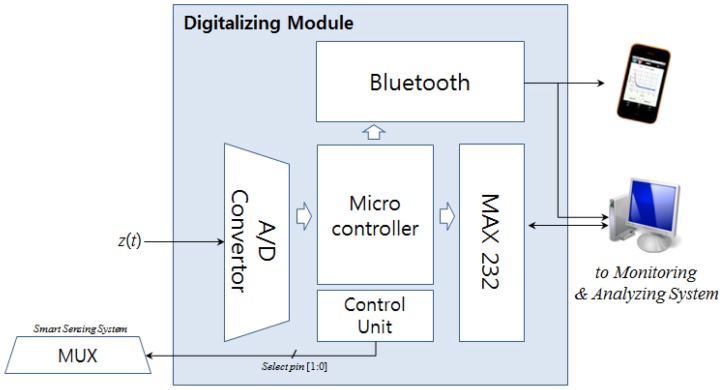
Structure of digitalizing module.

As shown in Figure 4, *z(t)* passes from the previous stage, and is digitalized by the ADC. Presently, *z(t)* is being passed to the ADC mixed with other signals, and the signals are not distinguishable. However, because of the select-pin [1:0], which passes signals by the control unit, and operates regularly at fixed time intervals, the ADC can perform with reference to time.

### 3.4. Design of Monitoring and Analyzing Module

**Figure 5.**
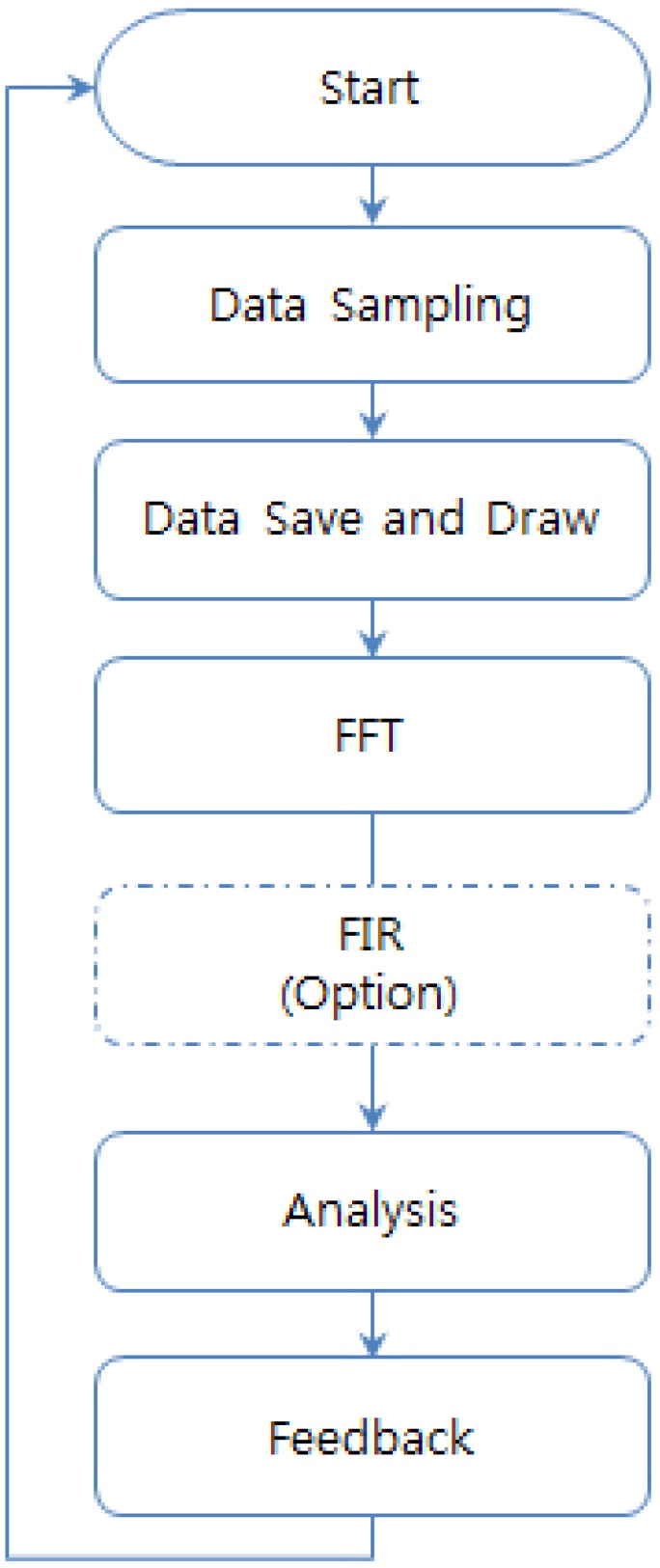
Flow chart of monitoring and analyzing system.

In this sub-section, the monitoring and analyzing module of the integrated monitoring and analyzing system is described. This part of the module consists of three sub-parts, as shown in Figure 5.

As seen in Figure 5, in the first step, the monitoring and analyzing module stores and draws signals that have undergone sampling. The next step consists of a Fast Fourier Transform (FFT) that converts signals in the time domain to equivalent signals in the frequency domain. The finite impulse response (FIR) removes the 60 Hz power-noise from the input signal. The final step in this module interprets and displays the obtained body signal information.

## 4. Experimental Setup

### 4.1. Participants

One right-handed graduate (male, 31 years of age) participated in the experiment. The participant did not suffer from any neurological disorders. He was a volunteer and participated in accordance with the Declaration of Helsinki. The participant was evaluated while he sat in a chair in a relaxed state.

### 4.2. Environment for Communication

**Figure 6.**
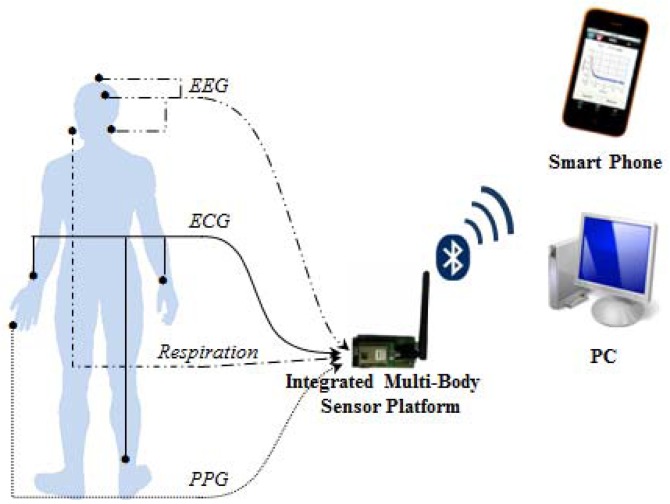
Environment for actual tests with integrated multi-body sensor platform.

Body-signals gathered using the integrated multi-body sensor platform were transferred to a smartphone and a PC through the Bluetooth module. As shown in Figure 6, we analyzed four amplitude frequency responses and four frequency quantities using the PC with a Bluetooth dongle.

**Figure 7.**
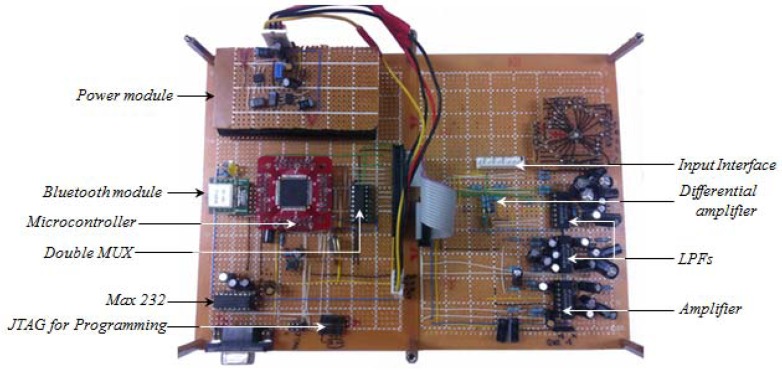
View of the prototype board.

As shown in Figure 7, we also saved the TX data from the MAX232 in the sensor platform, and compared it with the TX and RX data via Bluetooth communication on the PC.

### 4.3. Environment Power Consumption Checking

**Figure 8.**
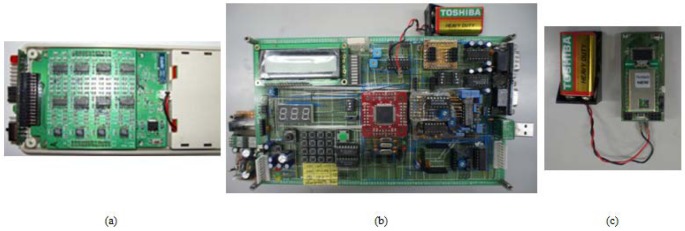
View of three type sensor platform for power consumption checking: (**a**) 8 channels EEG sensor platform using Zigbee communication; (**b**) Previous four channel multi-body sensor platform using Bluetooth communication; (**c**) Enhanced four channel multi-body sensor platform using Bluetooth communication.

The Figure 8(a) depicts the eight channel portable EEG sensor platform based on the 8051 core using Zibee communication (IEEE 802.15.4) built in 2009. Figure 8(b) depicts a four channel multi body sensor platform for measurement of EEG, ECG, Respiration and PPG using Bluetooth communication (IEEE 802.15.1). Finally, The Figure 8(c) is the four channel multi body sensor platform implanted with an enhanced design for the analog circuit using Bluetooth communication. The power consumption of each sensor platform was measured using a 9 V commercial battery.

## 5. Results

### 5.1. Frequency Responses

**Figure 9.**
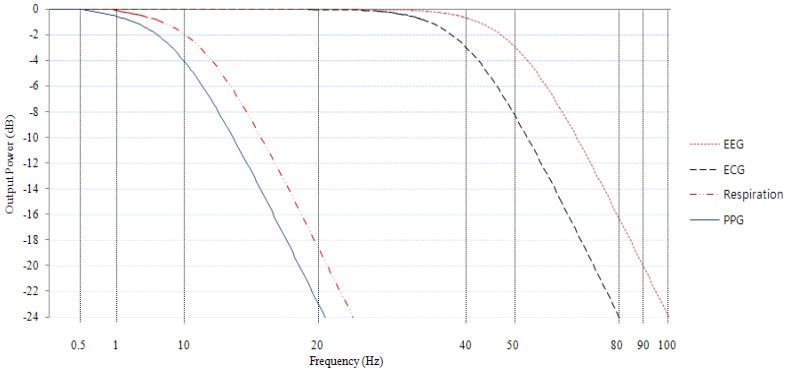
Amplitude frequency responses of low-pass filters inside multi sensing module.

Figure 9 shows the amplitude frequency responses (AFRs) of the LPFs inside the multi sensing module presented in this research. This result is calculated using a function generator (AFG310, Tektronix, Inc., Beaverton, OR, USA). Input frequencies between 0.01 Hz and 100 Hz were used to output a sinusoidal signal of amplitude 1 V; the signals were measured with an oscilloscope (DSO7012B, Agilent Technologies, Inc., Santa Clara, CA, USA). The results shown in Figure 9 confirm that each body signal underwent filtering in accordance with the LPF frequency ranges listed in Table 1. It has also been verified that the frequencies were 50 Hz for EEG, 40 Hz for ECG, 12 Hz for respiration, and that PPG fell below −3 dB at 8 Hz [13,14].

### 5.2. Frequency Contents

**Figure 10.**
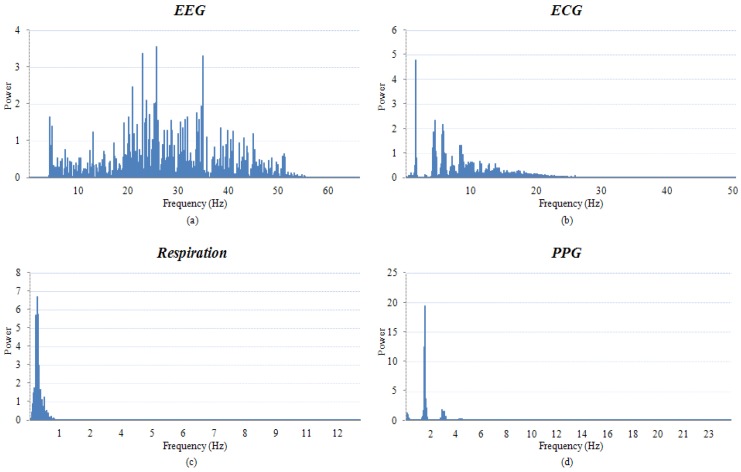
Fast Fourier-transform results (**a**) EEG frequency contents; (**b**) ECG frequency contents; (**c**) Respiration frequency contents; and (**d**) PPG frequency contents.

Figure 10 shows the frequency content of the various real body signal measured by the integrated multi-body sensor platform.

As illustrated in Figure 10(a), for EEG signals, frequencies between 4 Hz and 55 Hz were displayed, but signals below 4 Hz were filtered to avoid noise from the electrooculogram (EOG) signals. This represents the properties of the EEG signal very well. The frequency properties of the ECG signals in Figure 10(b) show that almost all the signals are located within 40 Hz. Although the LPF respiration range is below 12 Hz, most signals exist below 1 Hz, as verified by Figure 10(c). So, it seems desirable to narrow the LPF respiration range in future studies. The properties of the PPG signals shown in Figure 10 are typical of signals below 8 Hz.

### 5.3. Packet Reception Ratio

The payload of Bluetooth 2.0 is 2,864 bits per frame. Among them, the data packet payload is 2,746 bits per frame. In the standard for Bluetooth, the maximum data rate is 57,600 bits per second [15]. Therefore, it is possible to transmit approximately 20 frames per second [10]. The sampling rate of our sensor platform is 256 Hz and it has four channels. It has been programmed to transmit 2 Bps from the MCU to the Bluetooth module. Therefore, the data rate of our sensor platform is 16,384 bits. That is approximately 5.9 frames per second. In the real experiment through the PC with MAX232 in the prototype board (Figure 7), the packet reception ratio (PRR) was 99.99%. In the EEG sensor platform [Figure 8(a)], the average PRR of the Zigbee gateway was 99.5% [6].

### 5.4. Low Power-Consumption and Low-Cost System

**Figure 11.**
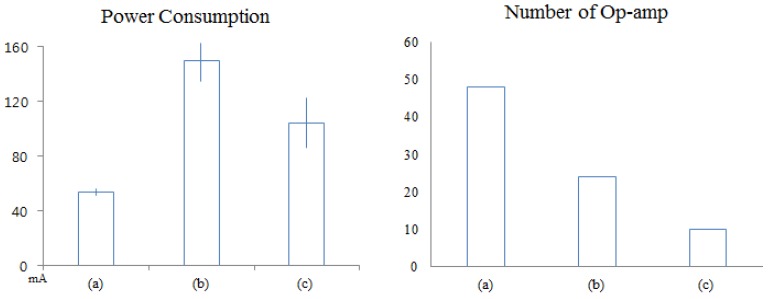
Comparison of power consumption (**left**) and Number of Op-amp (**right**): (**a**) 8 channels EEG sensor platform using Zigbee communication; (**b**) Previous 4 channels multi-body sensor platform using Bluetooth communication; (**c**) Enhanced 4 channels multi-body sensor platform using Bluetooth communication.

In Figure 11(a) the platform operates with eight channels using Zigbee communication. The average power consumption of this platform is 53.65 mA (The standard deviation (STD) is ±1.15 mA). The average power consumption of platform (b) is 149.4 mA (STD is ±12.5 mA). Finally, the average power consumption of the enhanced four-channel multi-body sensor platform is 104.1 mA (STD is ±18.3 mA).

In Figure 11(a) the eight channel EEG platform needs 48 op-amps, (b) platform needs 24 op-amps and (c) the enhanced multi-body sensor platform needs just 10 op-amps. These methods reduce the cost of the system and have an effect on the size and weight of the system.

### 5.5. A Real-Time Monitoring System

The dataset received through the Bluetooth dongle for a PC is viewed with the integrated multi-body sensor platform. In this program, the output waves can be saved in real-time and the output can be played, paused, and stopped. The embedded FIR allowed efficiently eliminating the power noise at 60 Hz [16].

**Figure 12.**
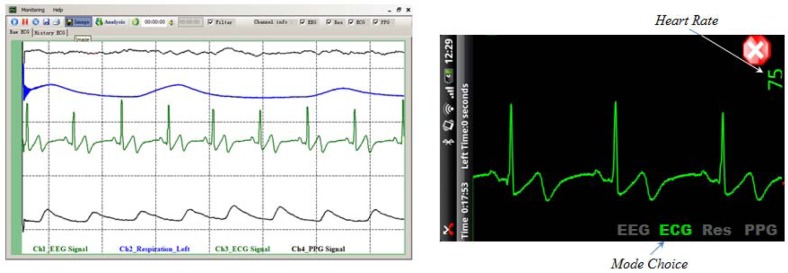
Screenshot of the integrated healthcare monitoring system (**on the left**) and of Smartphone application (**on the right**).

The left side of Figure 12 shows the waveform of the body signal measured by the integrated healthcare monitoring system. The right side of Figure 12 simply shows the ECG wave with a heart rate on the smartphone.

## 6. Conclusions

A single body-signal measured in a non-invasive way shows only a single aspect of a person's health. A human body has very intricate interconnected systems, and it is not appropriate to draw any specific conclusions from data obtained in this way. Therefore, in order to be able to draw more precise conclusions, various body-signals need to be measured simultaneously.

The goal of this research was to synchronize the measurement of various signals from the human body in a WBSN. This platform would contribute to the more accurate and synchronized measurement and analysis of changes in EEG, Respiration, ECG and PPG.

In WBSN, low power consumption requirement is very important. However, if too much emphasis is placed on low power consumption, it is difficult to satisfy the other requirements. Therefore, in order to create an efficient platform, it is necessary to have a balanced design and implementation.

The platform proposed in this paper is not the best solution in terms of power consumption. We did not use Zigbee communication, which consumes the least power among the available wireless communication methods. Our platform was instead designed for convenient use with smartphones and PCs via Bluetooth communication. Consequently, we were able to exclude the ZiGw gateway from our design. As a result, the overall cost could be reduced. However, we were faced with a new problem due to the Bluetooth communication in terms of power consumption. Nevertheless, we partially overcame it by using an enhanced analog circuit design. Moreover, due to the higher packet reception ratio (PRR), we did not require to implement within an interpolation algorithm anymore in the receiver.

In terms of innovation, as our platform can be used with smartphones and tablet PCs, synchronous multi-bio signals can be easily identified in real time.

Contributions by applications can be summarized as follows:

- Measurement of the various body-signals synchronously
- Real-time-monitoring
- A low power-consumption, low-cost, ultra-compact, lightweight system as a result of an enhanced analog design for decreasing the amplifying chips

Such a synchronous multi-body sensor platform is expected to be very useful in telemedicine and emergency rescue scenarios. Furthermore, we expect that this system will be able to analyze the mutual effects between body signals.

## Figures and Tables

**Table 1.** Body-signal specifications.

**Part Name**	**CMRR Circuit**	**Differential Amplifier**	**Low-Pass Filter**	**Total Gain**
EEG	○	○	<50 Hz	80 dB
ECG	○	○	<40 Hz	60 dB
Respiration	x	x	<12 Hz	40 dB
PPG	x	x	<8 Hz	34 dB
